# An International System of Electro-Therapeutics

**Published:** 1895-03

**Authors:** 


*An International System of Electro-Therapeutics.* By HORATIO R. BIGELOW, M.D., and Thirty-eight Associate Editors. Philadelphia: THE F. A. DAVIS COMPANY. London: F. J. REBMAN. 1894.

This "System" is the work of thirty American, two British, two Canadian, and four French writers. It may be described as monumental, as it runs to 1126 pages, and is certainly a very large book to tackle. It is, however, intended mainly as a work of reference, and therefore quite rightly aims at completeness. The editor informs us in the preface that "each paper is a classic of itself:" obviously 1894 will be responsible for quite a respectable addition to the number of our classical writers.

Taking the book as a whole, we first remark in what a variety of cases we can, if we choose, apply electricity. It appears that every disease to which mankind is liable may be so treated, with the exception of the specific fevers; we may doubtless anticipate that this omission will ere long be rectified. The plan of the work has apparently been to put each division of the subject into the charge of some person eminent for his knowledge of that branch, and to give him a free hand in writing his article. Excellent as this plan is in most respects, it has some disadvantages. In the first place, there is apt to be considerable overlapping in articles treating of different branches of the same subject; and secondly, some articles are likely to be of disproportionate length. The second failing is not particularly noticeable in this work; but there is considerable repetition, adding unduly to the length of the book without any corresponding advantage. Each author has been allowed to describe his own particular set of apparatus; it would have saved space and the reader's time, and also conduced to clearness, if descriptions of all the apparatus had been given in one section, with some general help from the editor to aid selection.

At present the number of batteries and other instruments is somewhat bewildering, to say nothing of the claims of rival makers. Then each writer should have been kept strictly to his subject; for instance, it is superfluous, in the section on "Electro-Therapeutics of Diseases of the Lungs and Heart," for the writer to describe at length the symptoms and general treatment of asthma, especially as the value of electricity in this disease, judging from the writer's own statements, is somewhat dubious. So, again, the description of Graves's disease is too long. No one would be likely to refer to a work on Electro-Therapeutics for a general account of asthma or of Graves's disease. A critical review of Apostoli's treatment illustrates a defect of the opposite kind: the author ends with a lengthy description of what he calls the "rapidly-alternating sinusoidal-induced current," which has little connection with the main subject of the article, and would have been more in place in one of the earlier sections of the work.

Each paper as it stands reads well, if referred to for itself alone; and, speaking generally, the various writers take common-sense views, and their statements appear to us to be trustworthy. Of course all the articles are not of equal merit, but in the main they attain a high standard. Merely as a work of reference for each particular subject this "System" is well compiled; but the editor does not seem to have attempted to weld together the articles to form one continuous whole, and there is therefore, as we have said, a good deal of redundant matter, without which the book would be stronger.

The amount of space given to the treatment of diseases of women by electricity, and the number of articles—no less than ten—devoted to this subject, seem to indicate that this is at present, at any rate in America, the fashionable mode of treatment. We cannot criticise these communications at length. Dr. Kellogg, in his paper on the "Methods of Apostoli and others," insists upon the necessity of accuracy in diagnosis, and of due precautions in carrying out electrical treatment. Dr. Inglis Parsons gives a short, clearly-written article on the treatment of cancer of the uterus by electricity, in which the reader will find the necessary information as to the selection of the cases and method of procedure most likely to lead to success.

The sections on Galvanism and Faradism are both good, but rather long; the former especially is too diffuse, and the history of the subject, more especially that of Franklin's researches, though interesting and well-written, is perhaps too lengthy for a System of Electro-*Therapeutics*. The article on Electro-Physiology, which overlaps that on Galvanism somewhat, is one of the best in the book, and contains a particularly clear account of the controversy as to the true meaning of muscle- and nerve-currents. There is a highly interesting and well-written chapter on Animal Electricity, by Prof. Wesley Mills. A clear, succinct description of the methods of Electro-Diagnosis, by Dr. W. F. Robinson, containing the essential points of the subject, precedes an article on Cataphoresis or Anodal Diffusion, by Dr. Peterson, who seems to have obtained good results from the anodal diffusion of drugs, especially in the treatment of facial neuralgia by ten to twenty per cent. solutions of cocaine. Local anæsthesia is produced without any constitutional effects and relief from pain afforded for some hours. He finds it possible to administer exact doses in this manner.

The electrical treatment of diseases of the nose, naso-pharynx, pharynx, and larynx has received full justice in the capable hands of Dr. Sajous. There is a most readable and excellent contribution on Neuroses, by Dr. Morton Prince, which will well repay careful study, and an equally good section on Diseases of the Spinal Cord, by Dr. William J. Morton.

We have not been able to particularise the other contributions, but we believe that the reader who refers to them will find in each full information on the subject of which it treats. There is a very good and full index, and the book will no doubt find its place as a useful work of reference. The editor is to be congratulated on bringing what must have been a very laborious task to a successful conclusion. The illustrations are good and abundant.

